# Poster Session II A321 ACTIVATION OF THE ARYL HYDROCARBON RECEPTOR DECREASES VISCERAL HYPERSENSITIVITY

**DOI:** 10.1093/jcag/gwaf042.321

**Published:** 2026-02-13

**Authors:** Y Habibyan, A Mohan, C Baggio, M Defaye, C Altier, K Sharkey, Y Nasser

**Affiliations:** Medicine, University of Calgary Cumming School of Medicine, Calgary, AB, Canada; University of Calgary, Calgary, AB, Canada; Medicine, University of Calgary Cumming School of Medicine, Calgary, AB, Canada; University of Calgary, Calgary, AB, Canada; University of Calgary, Calgary, AB, Canada; University of Calgary, Calgary, AB, Canada; Medicine, University of Calgary Cumming School of Medicine, Calgary, AB, Canada

## Abstract

**Background:**

Despite achieving endoscopic remission, up to 60% of patients with Inflammatory Bowel Disease (IBD) experience chronic abdominal pain. The gut microbiota has emerged as a key regulator in the pathophysiology of chronic pain. Patients with IBD in remission display decreased fecal levels of microbial indoles. These indoles serve as ligands for the Aryl hydrocarbon receptor (AhR), expressed by nociceptors.

**Aims:**

To investigate the role of AhR receptors in visceral sensitivity using a mouse model of colitis in remission.

**Methods:**

To model colitis in remission, male and female C57BL/6 mice were given 2.5% dextran sulfate sodium (DSS) in their drinking water for 5 days (n = 33) or drinking water alone (n = 33) and recovered for 5 weeks. At week 5, visceral pain was evaluated by measuring the visceral motor reflex (VMR) to colorectal distention. For 3 consecutive days prior to VMR, mice received either GNF (n = 8 postcolitis, n = 8 controls), a synthetic AhR antagonist; indole-3-pyruvic acid (IPyA) (n = 8 postcolitis, n = 7 controls), a natural AhR ligand; a combination of GNF and IPyA (n = 8 postcolitis, n = 8 controls); or vehicle control (n = 9 postcolitis, n = 8 controls). The spinal cord was collected for cFos evaluation, a marker for neuronal activation.

**Results:**

Postcolitis male and female mice exhibited visceral hypersensitivity compared to control mice. Treatment with IPyA in male and female postcolitis mice attenuated visceral hypersensitivity. Inhibition of AhR by GNF blocked the antinociceptive effect of IPyA in both male and female postcolitis mice. Interestingly, treatment with GNF in male and female control mice led to visceral hypersensitivity similar to that of postcolitis groups. In males, the postcolitis mice had increased neuronal activation in the spinal cord, shown by cFos expression. IPyA treatment reduced neuronal activation in the spinal cord of postcolitis males comparable to the control males. Postcolitis males treated with GNF and IPyA had increased neuronal activation in the spinal cord compared to IPyA postcolitis males.

**Conclusions:**

These findings indicate that inhibition of AhR signaling is pronociceptive in both male and female mice. Selective activation of AhR reduces visceral hypersensitivity and neuronal activation in the spinal cord of postcolitis male and female mice.

**Funding Agencies:**

CAG, CIHRTRIANGLE

